# Report of abnormal tail regeneration of *Eremias yarkandensis* (Sauria: Lacertidae) and its locomotor performance

**DOI:** 10.1002/ece3.11074

**Published:** 2024-03-01

**Authors:** Tao Liang, Jiang‐miao Ran, Qian‐ru Liang, Lin Leng, Jiang‐hao Du, Jia Wang, Lei Shi

**Affiliations:** ^1^ Xinjiang Key Laboratory for Ecological Adaptation and Evolution of Extreme Environment Biology, College of Life Sciences Xinjiang Agricultural University Urumqi China; ^2^ School of Zoology Tel Aviv University Tel Aviv Israel

**Keywords:** bifurcated, caudal autotomy, endurance, lizards, sprint speed

## Abstract

Caudal autotomy is a phenomenon observed in many reptile taxa, and tail loss is a pivotal functional trait for reptiles, with potentially negative implications for organism fitness due to its influence on locomotion. Some lizard species can regenerate a lost tail, which sometimes can lead to the development of more than one tail (i.e., abnormal tail regeneration) in the process. However, little is currently known about the impact of abnormal tail regeneration on locomotor performance. In this study, we document abnormal tail regeneration in *Eremias yarkandensis*, a reptile species native to northwestern China. Additionally, we investigated the sprint speed and endurance performance of these lizards. This study provides the first report on abnormal tail regeneration and its locomotor performance on a Chinese reptile. We suggest that the abnormal regeneration of tails may contribute to the accumulation of food reserves in the species. In light of our findings, we propose that herpetologists continue to share their sporadic observations and assess the locomotor performance of species experiencing abnormal tail regeneration, further expanding our understanding of this intriguing phenomenon.

## Graphical abstract

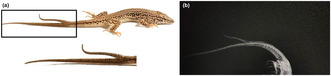

## 1 INTRODUCTION

The tail is a pivotal functional trait in reptiles, including lizards. It plays a crucial role in various aspects of their lives, such as visual displays, agonistic behavior, nutrition reserves (Chapple & Swain, 2002; Doughty et al., 2003), locomotor performance (Cromie & Chapple, 2012), and anti‐predation strategies (Lin et al., 2017). Notably, caudal autotomy, the voluntary shedding of the tail, is a widespread phenomenon in reptile taxa (Bateman & Fleming, 2009; Crnobrnja‐Isailović et al., 2016; Etheridge, 1967; Henle & Grimm‐Seyfarth, 2020; Moura et al., 2023), which distracts predators, thereby helping the reptile avoid becoming prey.

Tail loss, through caudal autotomy, offers an immediate survival advantage. Moreover, in many lineages, such as Lacertidae, Gekkota, and Scincidae, reptiles possess the remarkable ability to regenerate their tails after such losses (Arnold, 1984; Etheridge, 1967). However, not all tail regeneration events follow the typical pattern. Abnormal caudal regeneration can result in the growth of additional tails (i.e., more than one tail forms following caudal autotomy) in reptiles. This unusual phenomenon has attracted significant interest in recent years, with reports of it occurring in over 100 species of reptiles (see review in Barr et al., 2020). While some studies have investigated the abnormal regeneration process itself (Alibardi, 2021; Alibardi & Meyer‐Rochow, 1989; Lozito & Tuan, 2017), none have thoroughly explored the ecological factors driving this anomalous tail regeneration, although a study made significant progress in this direction (see Hayes et al., 2012).

Tail morphology significantly influences locomotor performance, and the loss of a tail in reptiles is negatively correlated with their locomotor abilities and stability (Hsieh, 2016). This, in turn, can impact various aspects of their life history, including climbing, jumping, habitat utilization, and ultimately, their prospects for survival (Jusufi et al., 2008; Kuo et al., 2012; Libby et al., 2012). It is highly conceivable that abnormal tail regeneration, with its associated increase in tail weight and altered morphology, might also have a negative impact on the kinematics of an individual's locomotion. Nonetheless, studies documenting the effects of abnormal tail regeneration on these life‐history traits remain scarce (Barr et al., 2020; Hayes et al., 2012), warranting further research.

While reports of abnormal tail regeneration span the globe, particularly in regions like America, Australia, and Europe, they have been under reported in Africa and Asia (Barr et al., 2020). To the best of our knowledge, there are few documented cases of abnormal tail regeneration from China (Barr et al., 2020; Xu & Zhu, 2023). During a recent herpetological survey fieldwork, we had the opportunity to capture 10 individuals of *Eremias yarkandensis* from northwestern China. Among these, abnormal tail regeneration was observed in one individual exhibiting two tails. To comprehensively understand the implications of this phenomenon, we conducted laboratory experiments to assess the locomotor abilities of these lizards. In this report, we present our findings on abnormal tail regeneration and its locomotor performance of *E. yarkandensis*.

## 2 MATERIALS AND METHODS

### 2.1 Study site and morphological measurement

We captured a total of 10 *E. yarkandensis* individuals (2 males, 8 females) from two closely located sites (85.5403° N, 37.4132° E, WGS 84, 2498 m a.s.l.; 85.7586° N, 37.4892° E, WGS 84, 2376 m a.s.l.) in Qiemo County, Xinjiang, China, on August 12, 2023, between 10:30 a.m. and 12:30 p.m. These specimens were transported to Xinjiang Agricultural University, where they were housed individually in plastic cages. Morphological traits, specifically snout‐vent length (SVL) and tail length (TL) were measured following the protocol outlined by Zhao et al. (1999). All measurements were accurate to within 0.01 mm.

### 2.2 Locomotor performance measurements

Before conducting locomotor performance tests, we determined the preferred temperature range for each individual by creating a temperature gradient ranging from 0°C to 50°C, as per the methodology described in Zheng et al. (2020). The lizards were introduced to the temperature gradient track, allowing them to freely move to warmer or cooler areas. After 10 min, we recorded their body temperatures. The average preferred temperature was 33.6°C, within a range of 32.1–35°C. Accordingly, all lizards were preconditioned at 33.6°C in an incubator for subsequent testing. For locomotor performance measurements, a 1.2 m horizontal track was utilized, divided into six segments, each spanning 20 cm. Lizards were placed on one end of the track, and their tails were gently stimulated with a hairbrush. A successful trial was defined as a continuous run from one end to another without stopping (Zheng et al., 2020). Video footage of the runs was recorded using a digital camera (SONY HDR‐CX405, FPS: 50) and subsequently imported into Adobe Premiere CS6 software to calculate the time taken (in frames) and the average speed for each segment. The track surfaces were covered with substrates 2 mm in thickness, consisting of sand, soil, and gravel collected from the local environment. The tests were performed on all individuals across all three substrate types, and we conducted the tests only once per individual within substrates. The maximum sprint speed within the substrates for each individual was determined by selecting the highest speed achieved among the six segments. Furthermore, endurance performance was assessed using a circular track with an inner ring of 4 m and an outer ring of 5 m. Lizards were stimulated to run with a hairbrush, and the total time (in seconds) they spent running until they ceased to respond to stimulation was recorded (Tan et al., 2020). This test was conducted on each individual across the three substrate types. We provided only descriptive statistics regarding the tests that were conducted because of the high unbalance in sample size between specimens with and without bifurcated tails, it was not possible to perform statistical analysis to test for differences.

## 3 RESULTS

**FIGURE 1:**
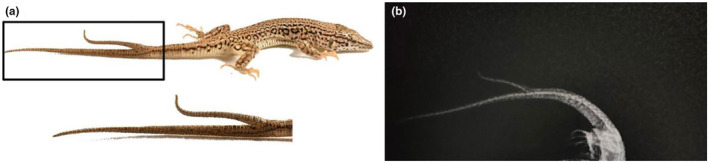
Individual of *Eremias yarkandensis* with bifurcated tail (a) and the X‐ray (b) from China. Photo by Tao Liang.

The point of bifurcation was located in the middle of the tail, towards the rear. The female *E. yarkandensis* exhibited an SVL of 48.40 mm, tail length of 63.32 mm, and a bifurcated tail measuring 19.2 mm in length, occurring within the middle third of the tail (Figure 1). For individuals without bifurcated tails, male SVL 51.57 ± 0.64 mm, female SVL 44.27 ± 1.51 mm (overall: 45.89 ± 1.58 mm). Male TL 73.91 ± 6.42 mm, female TL 68.23 ± 2.47 mm (overall: 69.49 ± 2.32 mm) (Table 1).

**TABLE 1:** Morphological and locomotor performance of *Eremias yarkandensis*.

Sex	SVL (mm)	TL (mm)	Sprint speed (m/s)			Endurance (s)		
			Sand	Soil	Gravel	Sand	Soil	Gravel
Male	50.93	80.33	1.42	1.67	1.67	57.94	74.34	95.06
Male	52.21	67.49	1.25	1.43	1.67	103.14	85.84	76.64
Female	48.23	65.41	0.90	1.43	1.25	114.54	161.36	81.64
Female	43.75	75.33	1.25	1.25	1.25	77.38	74.28	81.5
Female	42.73	63.61	1.25	1.18	1.41	NA	80.49	58.87
Female	48.4	63.32	1.43	2.00	1.82	60.42	82.98	74.2
Female	40.11	74.84	1.42	1.43	2.00	56.9	52.43	63.8
Female	39.06	70.94	1.67	1.25	1.41	54.64	53.85	40.13
Female	46.4	70.27	1.25	1.67	1.41	60.95	80.4	105.48
Female^a^	49.66	57.21	1.25	1.25	1.25	48.17	67.96	88.66

Abbreviations: SVL, snout‐vent length; TL, tail length.

^a^ Individual with bifurcated tail.

The female with the bifurcated tails displayed 2.00 m/s (soil), 1.82 m/s (gravel), 1.43 m/s (sand) in the sprint speed. While for the endurance, the female with bifurcated tail ran 60.42, 82.98, 74.2 s, in sand, soil, and gravel substrates, respectively. For the individuals without bifurcated tails, males showed 1.54 ± 0.11 m/s (soil), 1.66 ± 0 m/s (gravel), 1.33 ± 0.08 m/s (sand) sprint speeds, females showed 1.34 ± 0.06 m/s (soil), 1.43 ± 0.1 m/s (gravel), 1.28 ± 0.08 m/s (sand) sprint speeds. For endurance, males ran 80.09 ± 5.75 s (soil), 85.85 ± 9.21 s (gravel), 80.54 ± 22.6 s (sand), females ran 81.53 ± 13.99 s (soil), 74.29 ± 8.16 s (gravel), 68.76 ± 9.99 s (sand) (Table 1).

## 4 DISCUSSION

Incomplete autotomy events may result in abnormal tail occurrences if the original tail is retained. Indeed, such abnormalities might not be as rare as previously thought, even during the tail regeneration process (Sales & Ferire, 2019). However, comprehensive studies examining the potential ecological consequences of abnormal tails are notably lacking, despite numerous studies on the effects of tail loss. To our knowledge, this is the first study to provide comparative data on locomotor performance between specimens with and without abnormal tails (also see Xu & Zhu, 2023).

Abnormal tail regeneration is widely thought to increase an individual's mass, potentially altering their locomotor kinetics, which could, in turn, reduce a species' overall fitness in the field. Without enough sample size for individual with bifurcated tails, we can not explore its potential effects on locomotor performance. In the field, the female was observed basking at the entrance of a burrow and promptly retreated to her burrow upon detecting our presence, indicating that the abnormal tail probably did not impede her mobility. Besides, she had a relatively small bifurcated tail with little mass, which may have not adversely affected immediate survival, particularly in terms of sprint speed. Without any apparent disadvantages stemming from abnormal tail regeneration, it is highly conceivable that lizards may derive benefits from having multiple tails. Having a multifurcated tail might increase the chances of deflecting a predatory attack towards the tail, providing a larger target (Barr et al., 2019). On the other hand, during laboratory observations, the abnormal tail began to atrophy after a few days without regular food intake, whereas the original tail did not exhibit atrophy. Importantly, tails in reptiles can serve as storage for nutrition, potentially accumulating food reserves during times of high food availability and thereby increasing resistance to starvation. However, this aspect requires further investigation.

It is important to acknowledge that our understanding of how abnormal tails might influence the life‐history aspects of reptiles remains limited due to the scarcity of samples and studies. In this report, we only had one individual with an abnormal tail, which may not provide sufficient statistical power to detect potential ecological effects. Nonetheless, obtaining large sample sizes of individuals with abnormal tails in the field can be challenging. Therefore, we encourage the continued reporting of sporadic observations and their associated locomotor performance data to accumulate knowledge for future research. Alternatively, future studies could consider experimental manipulations involving artificial bifurcated tails (Barr et al., 2020).

## AUTHOR CONTRIBUTIONS

**Tao Liang:** Conceptualization (equal); data curation (equal); formal analysis (equal); investigation (equal); methodology (equal). **Jiang‐miao Ran:** Data curation (equal); formal analysis (equal); methodology (equal). **Qian‐ru Liang:** Data curation (equal); methodology (equal); software (equal); writing – original draft (equal); writing – review and editing (equal). **Lin Leng:** Data curation (equal); investigation (equal). **Jiang‐hao Du:** Data curation (equal); formal analysis (equal). **Jia Wang:** Data curation (equal); investigation (equal). **Lei Shi:** Conceptualization (equal); data curation (equal); formal analysis (equal); funding acquisition (equal); investigation (equal); methodology (equal); project administration (equal).

## CONFLICT OF INTEREST STATEMENT

The authors hereby state that there is no conflict of interest.

## Supporting information


Appendix S1

## Data Availability

All data that used in this note can be found in the supporting information (Appendix S1).
